# Molecular Hydrogen Affords Similar Neuroprotection to Therapeutic Hypothermia in a Porcine Model of Neonatal Hypoxic–Ischemic Encephalopathy

**DOI:** 10.3390/antiox14121405

**Published:** 2025-11-25

**Authors:** Emma Balog, Gábor Remzső, Valéria Tóth-Szűki, Éva Rózsa, Viktória Kovács, Ferenc Domoki

**Affiliations:** 1 Department of Physiology, Albert Szent-Györgyi Medical School, University of Szeged, 6720 Szeged, Hungary; 2 Centre of Excellence for Interdisciplinary Research, Development and Innovation of the University of Szeged (IKIKK), 6720 Szeged, Hungary

**Keywords:** hypoxic-ischemic encephalopathy, perinatal asphyxia, therapeutic hypothermia, molecular hydrogen, piglet model

## Abstract

Neonatal hypoxic–ischemic encephalopathy (HIE) remains a major cause of neonatal mortality and long-term disability, despite therapeutic hypothermia (TH) treatment, underscoring the need for further preclinical research. In the present study, we compared the neuroprotection afforded by TH and inhaled molecular hydrogen (H_2_) treatment in a translational newborn pig HIE model. Following 20 min of asphyxia induced by a hypoxic/hypercapnic gas mixture, piglets were reoxygenated and monitored for 48 h. Animals were randomly assigned to normothermia, continuous H_2_ ventilation (2.1%), or TH (33.5 °C for 37 h followed by slow rewarming) groups. Physiological parameters, electroencephalography (EEG), visual evoked potentials (VEPs), and neuropathology were assessed. TH eliminated post-asphyxia seizures and improved VEP latency, while H_2_ delayed seizure onset and increased quantitative EEG markers of signal complexity. Neuropathology revealed severe thalamic injury in normothermic controls, which was significantly attenuated by both H_2_ and TH, while neocortical, hippocampal, and basal ganglia injury was less extensive and not significantly altered by either of the neuroprotective interventions. These findings demonstrate that continuous H_2_ inhalation provides neuroprotection in HIE comparable to TH, particularly in the thalamus. H_2_ also exerts distinct electrophysiological effects, suggesting its therapeutic potential as a treatment for neonatal HIE.

## 1. Introduction

Neonatal hypoxic–ischemic (HI) encephalopathy (HIE) represents a severe neurological condition occurring in approximately 3 per 1000 live births in high-income countries, with even higher prevalence reported in less-developed regions [1]. At the cellular level, a critical pathomechanism of HIE is the imbalance of energy homeostasis usually triggered by decreased cerebral blood flow and oxygen supply. This disruption ultimately leads to several pathological processes, including oxidative stress, neuro-inflammation, and excitotoxicity, which all exacerbate neuronal injury [2]. The consequences of HIE can be profound and enduring, resulting in severe long-term neurological impairments such as cerebral palsy, intellectual disabilities, behavioral disorders, and epilepsy [3]. Furthermore, the extent of these complications of HIE is contingent upon both the severity of the initial injury and the interventions implemented during treatment [4]. This highlights the crucial importance of early diagnosis of HIE and the rapid implementation of effective therapeutic interventions to prevent the adverse outcomes associated with HIE.

In clinical practice, therapeutic hypothermia (TH), the controlled cooling of the neonate, was shown to reduce both HIE mortality and the incidence of long-term disabilities, and has become the standard treatment for HIE [5]. TH’s effectiveness is time-sensitive—with optimal neurodevelopmental outcomes achieved when the intervention is initiated within 6 h of the HI insult [6]. Although TH considerably improves survival rates after moderate to severe HIE, there is still a substantial residual mortality and morbidity [7]. Therefore, although TH is the only currently recognized neuroprotective HIE therapy, various additional treatment strategies including putative neuroprotective drugs, stem cell therapy, and neuroprotective gases are also undergoing laboratory testing [8]. Consequently, there is a critical demand for the development of additional therapeutic strategies for HIE that, either in combination with TH or as standalone interventions, could further reduce the incidence of mortality and severe complications.

In recent years, inhaled molecular hydrogen (H_2_) has gained prominence due to its neuroprotective qualities. In a seminal paper H_2_ protected neuronal cells from oxidative damage by targeting and reducing reactive oxygen species (ROS), demonstrating selective antioxidant properties [8]. Numerous animal models, both in vivo and in vitro, have shown that H_2_ has a neuroprotective effect. In a newborn piglet model, H_2_ significantly reduced oxidative markers and preserved neuronal integrity [9], as well as prevented delayed neurovascular unit dysfunction [10]. H_2_, when combined with TH, reduced seizures occurring after asphyxia in newborn piglets [11]. By preventing neuronal apoptosis, inhaled H_2_ has been shown to dramatically lessen post-HIE neuronal damage in the cortex and hippocampus in a rat model [12].

Based on the concept that H_2_ primarily functions as an acute antioxidant during early reperfusion, our group’s earlier research utilized a 4 h inhalation of H_2_ following neonatal asphyxia [9,10]. However, accumulating evidence suggests that H_2_ can also ameliorate more delayed neuronal injury pathways, including inflammation and apoptosis [12], indicating that its therapeutic window might be broader than previously thought. The present study specifically sought to determine whether extending H_2_ inhalation into the more delayed period of HIE development offered protection comparable to, or greater than, that provided by TH, the current standard of care.

## 2. Materials and Methods

### 2.1. Ethical Approval

All experimental procedures were conducted in accordance with the approval of the National Animal Committee on Animal Experiments (ÁTET, I.74–7/2015). The acquisition of animals was authorized by the National Food Chain Safety and Animal Health Directorate of Csongrád County, Hungary (permit number: XIV./1414/2015). All animal experiments were carried out in accordance with the ARRIVE 2.0 guidelines and the EU Directive 2010/63/EU concerning the protection of animals used for scientific purposes, as well as the guidelines established by the Scientific Committee of Animal Experimentation of the Hungarian Academy of Sciences and current Hungarian legislation on animal welfare (Government Decree 40/2013. (II.14.)).

### 2.2. Animals

On the morning of the experiments, newborn (postnatal day 1, <24 h old) male Landrace piglets (n = 22; body weight: 1790 ± 260 g) were obtained from a local supplier (Pigmark Co., Ltd., Szeged, Hungary). Only male animals were included in this study to minimize sex-related variability, as male piglets were shown to be more vulnerable to HIE than females [13]. Sodium thiopental (45 mg/kg; Sandoz, Kundl, Austria) was injected intraperitoneally to induce general anesthesia. The piglets were mechanically ventilated using a pressure-controlled small animal respirator that delivered warmed, humidified air (21% O_2_, balance N_2_), with the option of oxygen enrichment, after undergoing tracheotomy for intubation. Ventilation parameters were set between fraction of inspired oxygen (FiO_2_) 0.21–0.25, respiratory rate 30–32 breaths per minute, and peak inspiratory pressure 120–140 mmH_2_O in order to maintain physiological blood gas levels and proper oxygenation. In order to administer anesthetics, analgesics, and maintenance fluids, a catheter was placed into the right femoral vein under aseptic conditions. Morphine (100 μg/kg; Teva, Petach Tikva, Israel) and midazolam (250 μg/kg; Torrex Pharma, Vienna, Austria) were administered as an initial bolus. Morphine (10 μg/kg/h), midazolam (250 μg/kg/h), and isotonic fluids (5% glucose, 0.45% NaCl at 3–5 mL/kg/h) were then continuously infused. To continuously measure heart rate (HR) and mean arterial blood pressure (MABP), a second catheter was inserted into the right carotid artery. Servo-controlled heating/cooling pads with water circulation (Blanketrol III, Cincinnati Sub-Zero, Cincinnati, OH, USA) were used to continuously monitor and maintain the rectal temperature within the physiological range (38.5 ± 0.2 °C). Oxygen saturation (spO_2_), MABP, and HR were continuously monitored with an EDAN Im8 Vet Monitor (Edan Instruments Inc., Shekou, Nanshan, Shenzhen, China). Penicillin (50 mg/kg every 12 h; Teva, Petach Tikva, Israel) and gentamicin (2.5 mg/kg every 12 h; Sanofi, Paris, France) were given intravenously as preventive antibiotics. Every twelve hours, a suprapubic puncture was performed to empty the bladder. In order to ensure that physiological parameters were maintained during the observational period, arterial blood gas analysis, blood glucose, and lactate measurements (~200 μL per sample; EPOC Blood Analysis, Epocal Inc., Ottawa, ON, Canada), at baseline as well as at predetermined intervals up to 47 h were carried out.

### 2.3. Experimental Protocol

#### 2.3.1. Experimental Groups

**Figure 1:**
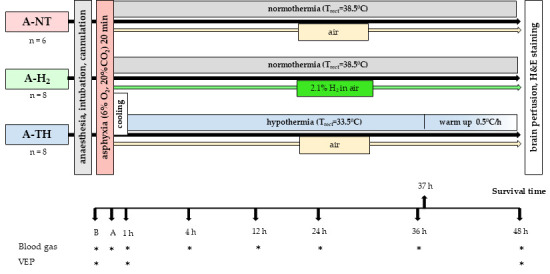
Graphical representation of the experimental protocol. As a control group, we used asphyxiated animals whose body temperature was maintained within the physiological range (38.5 ± 0.2 °C) (A-NT) throughout the 48 h observation period. H_2_ was inhaled at a concentration of 2.1% in air starting immediately after asphyxia (A), while maintaining normothermic body temperature (A-H_2_). In the third group, TH was initiated immediately after asphyxia. The animals were cooled to 33.5 ± 0.2 °C in 45–50 min, and TH was maintained until the 37th hour post-asphyxia, followed by rewarming at a rate of 0.5 °C/h for 10 h; thus, the animals regained physiological body temperature 1 h before the end of the observation period (A-TH). Arterial blood sampling and visual evoked potential (VEP) recordings were conducted at the designated time points (*).

After allowing stabilization of the observed physiological parameters for an hour after surgery, baseline physiological parameters were recorded. Three distinct experimental groups of animals were created (Figure 1). (1) Group asphyxia-normothermia (A-NT) (n = 6). (2) Asphyxia-H_2_-treated group with normothermia (A-H_2_) (n = 8). (3) Group treated with asphyxia-hypothermia (A-TH) (n = 8).

After baseline values were recorded, the animals were subjected to 20 min asphyxia, which was induced ventilating with a special hypoxic/hypercapnic gas mixture consisting of 6% O_2_, 20% CO_2_, and 74% N_2_ and simultaneously halving the respiratory rate (RR). The intravenous administration of glucose was also halted during the insult. After asphyxia, the animals were reventilated with air, except in the A-H_2_-treated group, where animals were reventilated with a special gas mixture consisting of 2.1% H_2_, 21% O_2_, and 76.9% N_2_. The RR and the intravenous glucose administration were also stabilized. Rectal body temperature was kept within the physiological range (38.5 ± 0.2 °C) in both the A-NT and A-H_2_ groups. In contrast, the animals of A-HT group were subjected to whole-body cooling starting coincidentally with the reventilation. These piglets achieved the TH, temperature of 33.5 ± 0.2 °C in 45–50 min after asphyxia. TH was maintained for 36 h; from the 37th post-asphyxia hour, the body temperature was progressively raised by 0.5 °C/h to rewarm the animals to normothermia one hour before the end of the observation period. Throughout the observation period, pressor therapy was given if needed to maintain the MABP over 40 mmHg with intravenous dopamine (5–15 μg/kg/min). Based on the continuous monitoring of oxygen saturation and end-tidal CO_2_ levels as well as blood gas measurements, the respiratory settings were adjusted to provide adequate oxygenation and normocapnia; mucus was aspirated from the endotracheal tube if required.

#### 2.3.2. Electrophysiology

**Figure 2:**
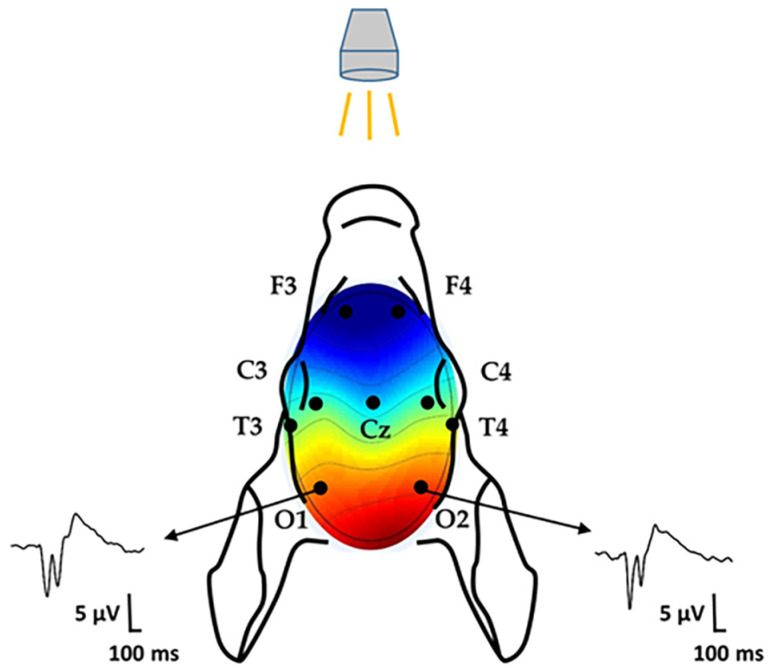
Top view of the modified neonatal EEG montage according to the international 10–20 system on the piglet scalp. Visual evoked potentials were recorded using a flashing stroboscope and the occipital leads.

Electroencephalography (EEG) activity was recorded using subcutaneously implanted silver electrodes placed above the fronto-parietal and occipital cortices, following the American Clinical Neurophysiology Society’s guidelines and employing a previously published eight-electrode montage covering fronto–parieto–centro–occipital regions (Figure 2) [14]. The signals were sampled at 256 Hz using a Nicolet One system (Natus Neurology, Middleton, WI, USA). Electrode impedance was regularly checked and maintained below 5 kΩ. EEG signals were amplified (Nicolet EEG v32, Natus Medical Inc., San Carlos, CA, USA), displayed online during the experiment, and saved for offline analysis.

For spectral analysis, the EEG signals were processed using custom MATLAB scripts, as described previously [14]. The broadband EEG was bandpass filtered (1–30 Hz) and decomposed into standard frequency bands (delta, theta, alpha, beta). Power spectral densities (PSDs) were estimated using Fast Fourier Transform (FFT) with a 30 s Gaussian window (kernel size 3–5) and Welch’s method. The PSDs were summed, averaged, and normalized to baseline.

As additional quantitative EEG markers, Spectral Edge Frequency (SEF) and Instantaneous Spectral Entropy (InstSpEnt) were calculated. SEF was defined as the frequency below which 95% of the total power resides in the 1–30 Hz range, and was computed for each EEG epoch and channel. This metric provides a summary measure of the frequency distribution and is sensitive to shifts in cortical activation and suppression, commonly used to evaluate the depth of anesthesia or neurological recovery. InstSpEnt was computed based on Shannon entropy to reflect the spectral complexity of the signal, with higher entropy indicating greater distribution of power across frequencies. Due to the continuous nature of the recordings and the use of narrow frequency windows, no segmentation was applied. Both SEF and InstSpEnt values were calculated for each EEG channel and presented separately for each treatment group. Additionally, Shannon entropy was also used as a feature for seizure detection. Seizures were defined as epileptiform EEG activity (spike, spike-and-wave, and oscillatory) that were often associated with motor activity presenting as tonic–clonic convulsions potentially affecting the mechanical ventilation. If seizures were detected, we attempted to alleviate them with an intravenous bolus of midazolam (250 μg/kg) and if ineffective, with an intravenous infusion of sodium thiopental (3.5 mg/kg/hour). The total duration of electrographic seizures was determined offline using spectral EEG analysis.

VEPs were elicited using 1 Hz flashes generated by a stroboscope, delivered in 10 trains of 10 stimuli each, with 10 s intervals between trains; during VEP recordings, ambient light was minimized. VEP waveforms were obtained by averaging 100 trials. The amplitude and latency of the P100 component were measured, and grand mean averages were calculated.

#### 2.3.3. Neuropathology

At 48 h after asphyxia, both carotid arteries of the anesthetized animals were cannulated in the distal direction, the chest was opened, the right atrium was cut then the head was perfused with cold (4 °C) physiological saline solution. The brain was then removed from the skull, and the two hemispheres were separated. The left hemisphere was immersed in 4% paraformaldehyde solution for fixation to be examined later.

After 2 weeks of fixation, the hemispheres were cut into blocks and embedded in paraffin. The 5 µm frontal sections prepared using a microtome (Leica Microsystems, Wetzlar, Germany) were mounted on silanized slides. The tissues were stained with hematoxylin–eosin (H&E). The neuropathological analysis of the CA1/CA3 subregions of the temporal/ventral hippocampus and the subcortical regions (the anterior portion of the caudate nucleus and the putamen, as well as the anterior and ventral anterior nuclei of the thalamus) commenced using a series of photomicrographs taken from each brain region. Injured and viable neurons were identified and counted by observers blinded to the experimental groups using the ImageJ software (version 1.53c, Wayne Rasband, NIH, Bethesda, MD, USA); the ratio of the damaged neurons were expressed as a percentage of the total neuronal count. The injured neurons were identified based on their appearance featuring a dark eosinophilic cytosol as well as pyknotic or damaged nuclei.

In the cerebral neocortex, neuropathology analysis were performed using a previously established neuronopathology score system described in detail here [10,15]. Briefly, the pattern of neuronal injury was evaluated in 40 non-overlapping visual fields from each cortical region at 20× magnification using light microscopy (Leica Microsystems, Wetzlar, Germany). The assessed neocortical regions included the medial and lateral prefrontal cortex (frontal region), the coronal gyrus, the cruciate gyrus (parietal region), the ectosylvian gyrus, the parahippocampal gyrus (temporal region), the posterior portions of the marginal gyrus, and the ectomarginal gyrus (occipital region). Neuronal injury severity in each visual field was categorized as follows: none < scattered < grouped/laminar < panlaminar. Each cortical region received a score (0–9) based on the frequency of the most severe injury pattern observed within the examined fields. The higher scores reflect more extensive and more severe neuronal damage. As we found no significant differences among the groups in any assessed cortical regions, the individual scores were summated and only these sums are presented in the Results.

#### 2.3.4. Statistical Analysis

All data are presented as mean ± standard error of the mean (SEM). Statistical analyses were performed using SigmaPlot (v12.0, Systat Software, Chicago, IL, USA) and MATLAB (version R2018b, MathWorks, Natick, MA, USA). A *p*-value less than 0.05 was considered statistically significant. Normality of data distribution was assessed using the Shapiro–Wilk test, and homogeneity of variances was checked with Levene’s test where applicable.

Physiological parameters, including HR, MABP, and blood gas values, as well as VEP amplitudes and latencies, were analyzed using two-way ANOVA to evaluate the effects of treatment and time, followed by Tukey’s multiple comparisons test. EEG-derived measures, such as InstSpEnt and SEF, were compared across treatment groups using one-way ANOVA with Tukey’s post hoc test. Neuropathological data, expressed as the percentage of damaged neurons in various brain regions, were evaluated using the Kruskal–Wallis test followed by Dunn’s multiple comparisons, due to non-normal data distribution.

## 3. Results

### 3.1. Physiological Parameters

**Figure 3:**
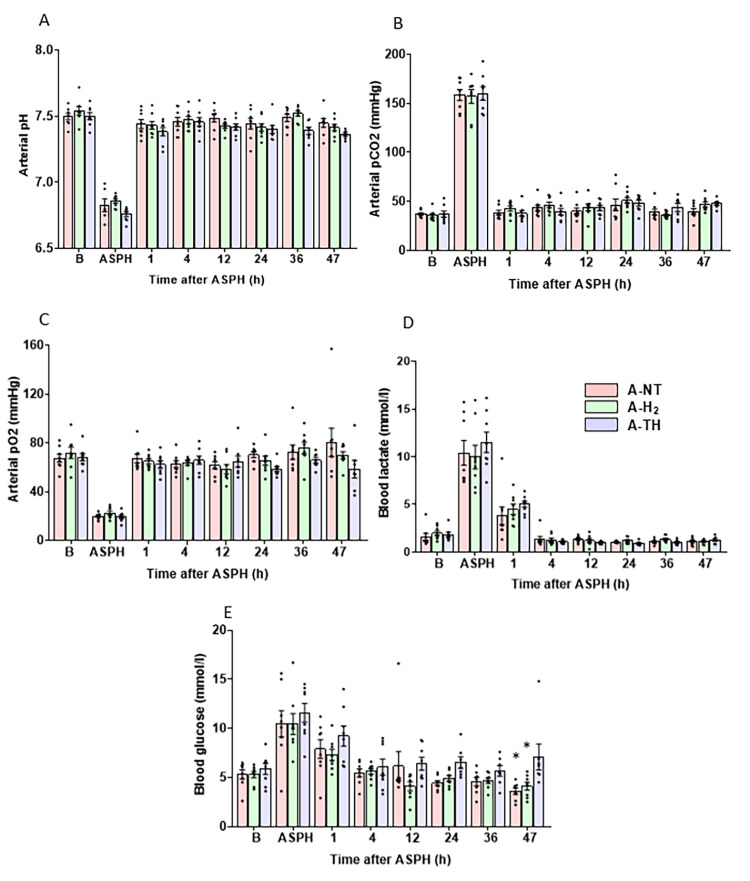
Blood chemistry data from arterial blood samples taken at baseline (B), the last minute of the 20 min asphyxia (ASPH), and 1–47 h after the start of the reventilation at six designated time points. At baseline (B), all values were within the physiological ranges, and the values of the three experimental groups were similar. Asphyxia triggered severe alterations in arterial pH (**A**), pCO_2_ (**B**) and pO_2_ (**C**) that were similar in the three experimental groups, the values returned toward the baseline by the first hour after asphyxia, and remained stable in each experimental group over the observation period. The blood lactate levels (**D**) showed large increases during asphyxia in all experimental groups without any significant differences among the study groups. Lactate levels were reduced gradually, showing still elevated levels at 1 h after asphyxia and returned to baseline levels at 4 h after asphyxia. Blood glucose levels (**E**) showed a similar course to lactate, there was a tendency of slightly higher glucose levels in the A-TH group, but this difference became significant only at the last time point. Data are presented as mean ± SEM. A-NT vs. A-TH * *p* = 0.002; A-H_2_ vs. A-TH * *p* = 0.004; n = 22.

**Figure 4:**
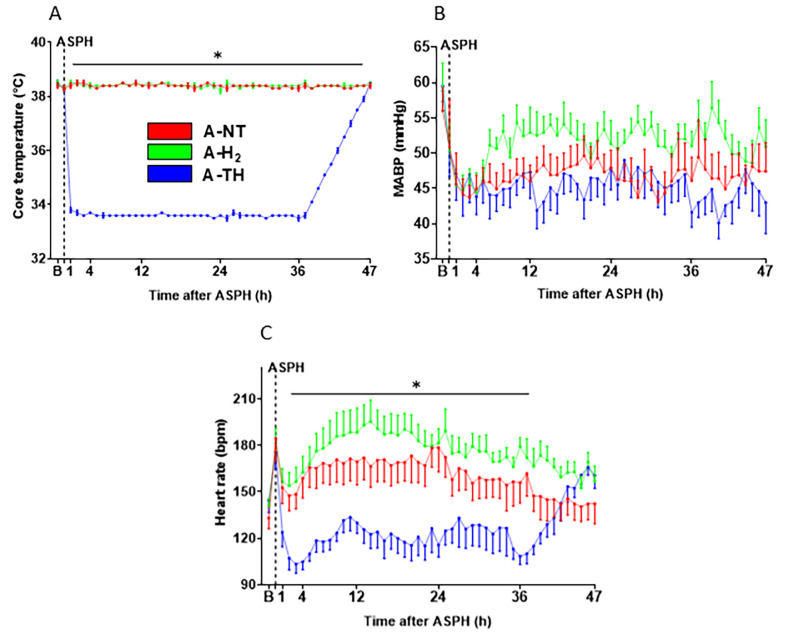
Body temperature and hemodynamic parameters. (**A**): The rectal temperature was maintained rigorously at 38.5 °C throughout the experiments in the A-NT and A-H_2_ groups. In the A-TH groups, the normothermic temperature was also maintained during asphyxia (ASPH), but immediately after asphyxia the animals were cooled to therapeutic 33.5 °C. From the 37th hour of the experiment onwards, the animals were continuously rewarmed by 0.5 °C/h. (**B**): MABP was maintained above the lower limit of cerebral blood flow autoregulation in all three groups, with no significant difference detected among the groups. (**C**): TH reduced HR in the A-TH group compared to the nomothermic groups; however, HR was restored toward baseline (B) values upon rewarming. Data are shown as mean ± SEM. Core temperature: A-NT vs. A-TH * *p* < 0.001; A-H_2_ vs. A-TH * *p* < 0.001; HR: A-NT vs. A-TH * *p* = 0.006; A-H_2_ vs. A-TH * *p* < 0.001; n = 22.

At baseline, before the onset asphyxia, the monitored blood chemistry (Figure 3) and other physiological parameters (Figure 4) were within their respective physiological ranges, and there was no significant difference among the three experimental groups. Asphyxia resulted in severe hypoxia and combined respiratory/metabolic acidosis (Figure 3): the drops in pO_2_ and pH, as well as the rises in pCO_2_, lactate, and glucose levels were similar in magnitude indicating similar insult severity. After asphyxia, most blood chemistry parameters were normalized by 1 h after asphyxia, except for the blood lactate levels that remained elevated at 1 h but were normalized at 4 h (Figure 3D). There were no significant differences in the measured blood parameters over the whole observation period except that blood sugar levels were significantly higher in the A-TH group compared to the other two groups at the 47th hour. During the observation period after asphyxia, the rectal body temperature was rigorously maintained in the three experimental groups according to study protocol. The rapid cooling and the slow rewarming in the A-TH group is well-documented (Figure 4A). Throughout the experiment, MABP was kept above ~40 mmHg (Figure 4B). To achieve this goal, n = 2 in the A-NT, n = 1 in the A-H_2_, and n = 4 in the A-TH piglets required dopamine infusion as an inotropic agent. In these animals, the average total dopamine dose used amounted to 6.31 ± 5.55, 15.51 and 19.84 ± 11.9 mg, respectively. TH elicited the expected reduction in HR compared to the normothermic groups (Figure 4C) that was reversible upon rewarming.

### 3.2. Electrophysiology

**Figure 5:**
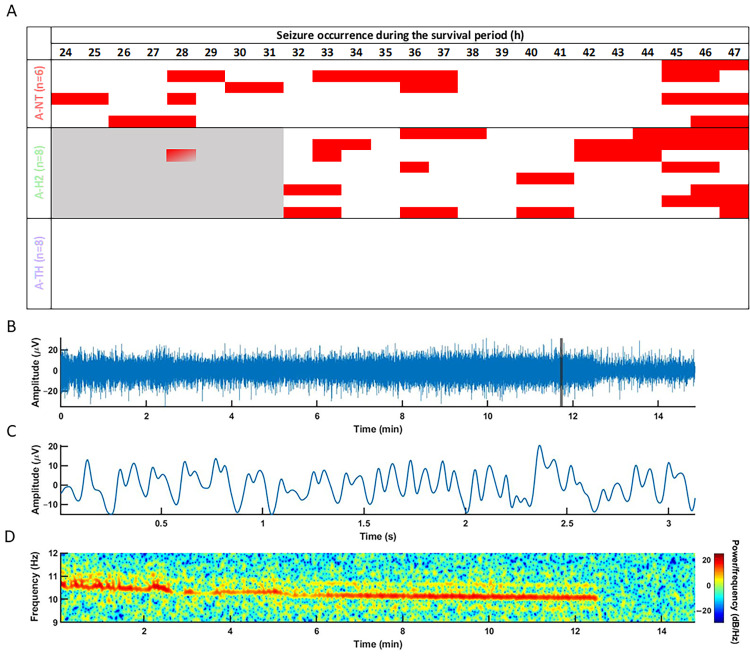
(**A**): Incidence of electrographic seizures on the second day of the observation period. Seizures were detected in all but one animal in the A-NT group, in every animal of the A-H_2_ group but none in the A-TH group indicating complete seizure suppression by TH. Interestingly, the onset of seizures was delayed in the A-H_2_ group compared to the A-NT group, only one seizure instance was recorded in the A-H_2_ group between the 24th and 32nd hours of the observation period (gray shaded area); n = 22. (**B**–**D**): representative seizure recording from a subject in the A-H_2_ group. (**B)**: Increased amplitude EEG signal during the approximately 13 min-long seizure episode. (**C**): Highlighted 3 s long spike-and-wave seizure waveform. (**D**): Representative spectrogram of a generalized seizure (10 Hz).

**Figure 6:**
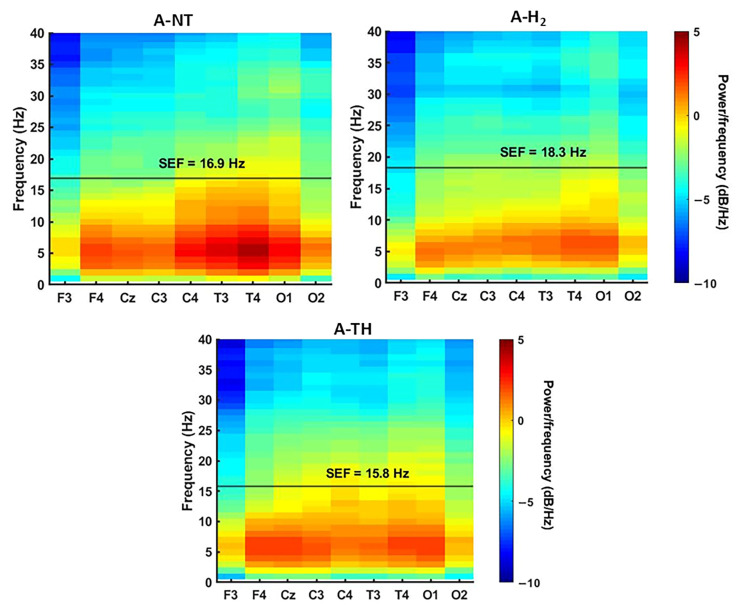
Representative PSD distributions of EEG recorded at the 47th post-asphyxia hour is shown for each experimental group. The curves represent averaged PSD across all animals within each treatment. Vertical lines indicate the SEF, with the highest value observed in the A-H_2_ group and the lowest in the A-TH group.

**Figure 7:**
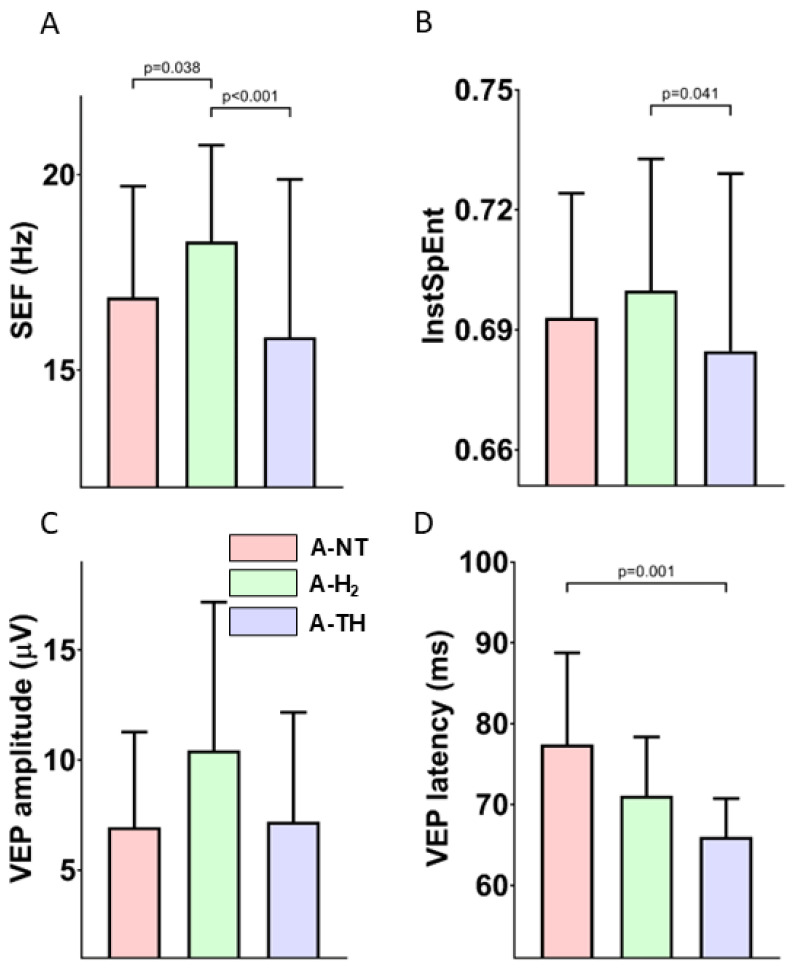
(**A**): The SEF values were significantly higher in the A-H_2_ group compared to both the A-NT and A-TH groups. No significant difference was observed between the A-NT and A-TH groups. (**B**): The bar graph shows the average InstSpEnt values across all EEG leads. The A-NT and A-H_2_ groups exhibit similar entropy levels, both significantly higher than those measured in the A-TH group, significantly different from the corresponding value of the A-H_2_ group. (**C**): There was no statistically significant difference in VEP amplitude between the experimental groups. (**D**): The VEP latency was significantly reduced in the A-TH group compared to the A-NT, while no significant change was observed for A-H_2_. n = 22.

Baseline EEG and VEP parameters were similar to previously reported values and were not significantly different among the experimental groups. Asphyxia quickly elicited isoelectric EEG that was gradually regenerated over the observation period in all animals. Electrographic seizures were detected usually after 24 h of reventilation (Figure 5A), with representative examples of seizure morphology, waveform, and spectral characteristics (Figure 5B–D). The total duration of seizure activity did not differ significantly between the A-NT (3.4 ± 1.0 h) and A-H_2_ (3.1 ± 0.7 h) groups (*p* = 0.81). Importantly, no seizures were observed in the A-TH group; in contrast, virtually all animals in the A-NT and A-H_2_ groups exhibited seizures. Interestingly, the onset of seizures was delayed in the A-H_2_ group compared to A-NT (Figure 5). By the end of the observation period, the PSD distribution of the EEG signal was similar in the three experimental groups with the low frequencies dominating the signal (Figure 6, Supplementary Figure S1), and SEF was significantly higher in the A-H_2_ group than in the A-TH group indicating a larger percentage of high frequency signal in this group (Figure 7A). Similarly, InstSpEnt was also higher in the A-H_2_ group signaling higher complexity in the signal despite the confounding presence of seizures (Figure 7B). VEP analysis at the end of the observation period revealed no significant differences in the amplitude of the P100 component; however, its latency was significantly shorter in the A-TH group as compared to the control values of A-NT (Figure 7C,D).

### 3.3. Neuropathology

**Figure 8:**
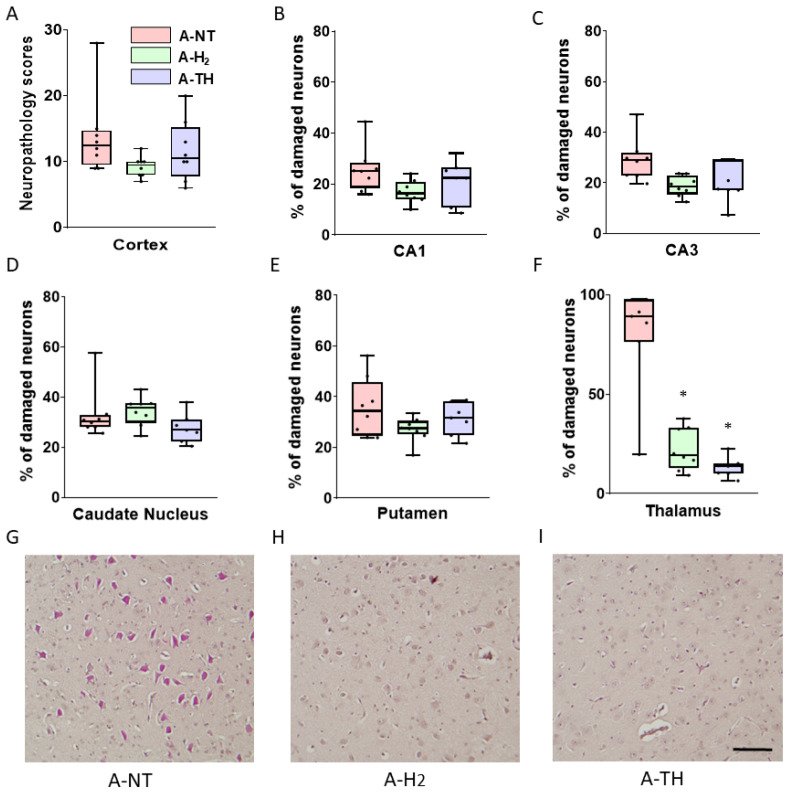
Neuropathology. Sum of neuropathological scores (**A**) of frontal, parietal, temporal and occipital neocortical areas. The cortical neuronal injury was minor; the neuropathological scores reflect sporadic neuronal injury in most assessed regions. There were no statistically significant differences among the groups. Neuronal injury was also modest in the CA1 (**B**) and CA3 (**C**) regions of the hippocampus in the A-NT group and there was no significant difference compared to either the H_2-_ or TH-treated groups. Similarly, no significant improvement was detected in the caudate nucleus (**D**) or the putamen (**E**) in either groups. In stark contrast, neuronal injury was near-complete in the thalamus (**F**) of the A-NT group, where both H_2_ and TH treatment significantly ameliorated the extent of neuronal damage. Representative photomicrographs of H&E-stained sections from the thalamus (**G**–**I**). Asphyxia-induced extensive neuronal injury in the A-NT group as indicated by the high proportion of damaged red neurons observed (**G**), while significantly less neuronal damage was detected in the A-H_2_ (**H**) and A-TH (**I**) groups. Scale bar: 100 μm. A-NT vs. A-H_2_ * *p* < 0.0001; A-NT vs. A-TH * *p* < 0.0001; n = 22.

Neuropathological examination revealed a low to moderate injury in most assessed brain regions. In the cerebral neocortex, neuropathological scores were similar, and there were no significant differences among the groups in any region. Therefore, the scores of the four cortical regions were summated and analyzed together, showing no significant differences among the three groups (Figure 8A). Similarly, the percentage of damaged neurons were low in the CA1 and CA3 regions of the hippocampus in the untreated A-NT group (Figure 8B,C) and the basal ganglia (Figure 8D,E), and consequently there was no discernible neuroprotection exerted by either H_2_ or TH. However, the neuronal injury in the thalamus was very severe in the A-NT group, and significant amelioration of the injury was detected in both the A-H_2_ and the A-TH group indicating neuroprotection (Figure 8F–I).

## 4. Discussion

The major novel finding of the present study is that continuous H_2_ inhalation during the acute phase of HIE development (for 48 h after asphyxia) affords neuroprotection comparable in magnitude to TH. More specifically, the marked asphyxia-induced neuronal injury in the thalamus was equally ameliorated by H_2_ and TH. In addition, both treatments benefited neuronal function shown by various electrophysiological parameters. Accordingly, we found that TH completely suppressed electrographic seizures and accelerated the recovery of VEP latency, while H_2_ treatment delayed seizure onset but also improved SEF and InstSpEnt, suggesting complementary neurophysiological actions.

The cerebroprotective actions of inhaled H_2_ in piglet HIE models have been reported previously. In particular, acute inhalation of H_2_ reduced oxidative stress markers, preserved the neuronal architecture, and lessened delayed neurovascular dysfunction in asphyxiated neonatal piglets [9,14]. Our previous studies employed a 4 h H_2_ inhalation regimen after asphyxia in accordance with the outcome measure time points at 4–24 h of HIE development, such as neurovascular reactivity, neuronal injury, or electrophysiological measurements [9,10,14]. However, in one previous work we tested if the 4 h H_2_ inhalation could augment the neuroprotective effect of TH. In that study, we induced asphyxia with a more severe hypoxic component as the hypoxic/hypercapnic gas mixture used for asphyxia induction contained 4% instead of 6% O_2_. However, all other aspects of the study protocol such as the 48 h observation period, anesthesia, mechanical ventilation, TH management, and supportive therapy were identical [14]. In that study, neuropathological examination revealed very severe neuronal injury in virtually all brain regions studied—injury was pronounced in the basal ganglia, thalamus, the hippocampus, and the neocortex. Furthermore, TH was unable to yield significant neuroprotection in virtually any region neither alone nor in combination with H_2_. Therefore, the present study combined the 48 h observation period with a milder hypoxic component (6% O_2_ vs. the 4% O_2_ in [14]). Although we expected milder neuronal injury, it was surprising that the neuropathological scores of the neocortex, and percentage of injured neurons in the hippocampus as well as the basal ganglia were found close to the normoxic time control values of the previous study. A clear limitation of the present study is that sham-control groups were not included; however, the present findings showing minimal neuronal damage in both treated groups suggest that TH or H_2_ treatment alone, in the absence of asphyxia, would not have likely compromised the neuronal viability as well. In contrast to most other examined regions, however, neuronal injury in the thalamus was pronounced in the present study as well, thus allowing the evaluation of neuroprotection of the treatments in this region known to be vulnerable to HI damage. Indeed, the thalamus is known to be affected in moderate and severe HIE in term newborns, and thalamic lesions can contribute to motor impairments [16]. We demonstrate in the present study that both H_2_ and TH exert significant neuroprotective effects in the thalamus; in fact, the percentage of injured neurons after asphyxia was reduced almost to the level of time controls from the previous study [14] by either treatment. However, the lack of detectable neuroprotection in the neocortex suggests that this mechanism may be spatially limited, and that the thalamic preservation alone was insufficient to prevent cortical structural injury under the present conditions. On one hand, this shows the remarkable neuroprotection exerted by H_2_ in this region; on the other hand, regrettably, the potential synergistic action between H_2_ and TH could not be tested.

Other research groups have also consistently demonstrated the neuroprotective potential of inhaled H_2_ in slightly different piglet HIE models. Htun et al. reported that combined H_2_ ventilation and mild hypothermia improved neurological scores, reduced seizure burden, and preserved cerebral energy metabolism outcomes 5 days after the asphyxic insult in a neonatal pig model [11,17]. Using the same experimental conditions they also showed that TH combined with H_2_ was significantly more effective than the TH alone 24 h after the asphyxic insult: cerebral blood volume increased, while cerebral hemoglobin oxygen saturation decreased [18]. More recently, Inoue et al. demonstrated that 6 h of H_2_ inhalation attenuated neuronal cell death, although H_2_ was unable to improve the initial EEG suppression [19]. These findings are in accordance with our previous studies showing not only the neuroprotection afforded by H_2_, but that its mechanism of action likely involves the suppression of asphyxia-induced pro-inflammatory cyclooxygenase-2 upregulation and microglia activation in the neocortex and hippocampus of newborn piglets, further supporting its potential to combat neuro-inflammation [20].

In rodent models of neonatal HI, H_2_ administration (either via inhalation or hydrogen-rich water gavage) significantly reduced neuronal damage in the hippocampus and cortex by inhibiting apoptotic processes and attenuating microglial activation [9,12,21]. Beyond neonatal HIE, beneficial effects of H_2_ have also been observed in models of neurodegeneration and toxic brain injury, further underscoring its potential as a broadly relevant neuroprotective strategy [22,23]. Therapeutic effects of H_2_ were noted in adult ischemic stroke and subarachnoid hemorrhage models too, with hydrogen inhalation or hydrogen-rich solution ameliorating oxidative damage, inhibiting blood–brain barrier breakdown, and improving neurological function [24,25,26]. Mechanistically, the beneficial effects of H_2_ have been attributed to its unique redox-modulatory activities. H_2_ selectively scavenges hydroxyl radicals and peroxynitrite, two of the most reactive oxygen/nitrogen species with the highest toxicity, without interfering with physiological redox signaling through nitric oxide or superoxide [24,25]. This allows for H_2_ to mitigate oxidative stress but preserve signaling pathways required for cell repair and survival. Aside from its antioxidant activity, H_2_ also initiates anti-apoptotic effects through the blockage of caspase-3 activation and modulation of major stress–response pathways [27,28]. Moreover, H_2_ inhibits microglial activation and the production of pro-inflammatory cytokines and therefore extinguishes the secondary neuro-inflammatory cascade [29]. The synergistic antioxidant, anti-apoptotic, and anti-inflammatory effects interrupt the feed-forward loop of secondary neuronal injury and ultimately preserve neuronal viability and facilitate functional recovery [30]. These findings are consistent with prior comprehensive reviews summarizing the diverse neuroprotective actions of H_2_ in neonatal and adult brain injury models [31]. Beyond neonatal models, the beneficial effects of H_2_ have also been demonstrated in other contexts of neurodegeneration and toxic injury [23,32,33], further supporting its potential as a broadly applicable neuroprotective agent. Given its favorable safety profile and preclinical evidence of neuroprotection, H_2_ may serve as a valuable pharmacological adjunct or as monotherapy in cases where TH is contraindicated within clinical settings [34,35].

Throughout the experiment, physiological parameters such as MABP, blood gases, and HR were kept within the target range according to the study protocol, ensuring comparable systemic conditions across groups. Interestingly, the need for vasopressor support appeared to have differed between treatments: dopamine administration was required more frequently in the A-TH group (four out of eight) compared to both normothermic groups, whereas H_2_-treated animals needed it least often (one out of eight). This observation is consistent with previous findings in the literature that TH induces bradycardia, thereby increasing the risk of hypotension and need for pharmacologic circulatory support, particularly during cooling and rewarming phases [36,37]. In contrast, the lower dopamine requirement in the H_2_ group suggests that H_2_ inhalation did not compromise cardiovascular function and may even confer some cardiovascular protective effects. Indeed, several studies have demonstrated that H_2_ mitigates ischemia–reperfusion injury in the heart, preserves myocardial function, and attenuates systemic inflammatory responses [38,39], raising the possibility that part of its beneficial effect observed in the present study could be mediated through improved hemodynamic stability. While TH also exerts neuro- and organ-protective effects, these are mediated through a combined effect of hypometabolism, reduced excitotoxicity, and modulation of inflammatory cascades. However, TH may also impair thermoregulation, delay drug metabolism, and increase the risk of arrhythmias and coagulation disturbances if not carefully managed [36,40,41].

Electrophysiologically, in our previous studies, we reported universally compromised EEG recovery with no considerable differences related to treatment, but here TH completely eliminated post-asphyxia seizures and decreased VEP latency, and H_2_ delayed seizure onset and increased quantitative EEG indicators (SEF and InstSpEnt) [14]. SEF depended on both the timing and intensity of seizure activity. Upward shifts occurred when a seizure emerged as a new component, but such seizure-related components were absent in TH-treated animals. Regarding VEPs, no abnormalities were observed in the overall VEP pattern, and all stimuli elicited responses in all of the experimental groups. We focused on the P100 component, which was consistently present in all groups, the only significant difference being a shorter latency in the A-HT group compared with the A-NT group. The marked thalamic protection observed in the present study indicates that H_2_ may act at the level of thalamo-cortical network integrity. As the thalamus plays a critical role in relaying and synchronizing cortical activity, preservation of thalamic neuronal viability could facilitate the restoration of large-scale cortical network function [42]. This interpretation is supported by the improved spectral EEG parameters (SEF, InstSpEnt) and faster VEP recovery observed in the H_2_-treated group. Mechanistically, it is also possible that suppression of oxidative stress and preservation of thalamic microcirculation by H_2_ contributed to the local neuronal protection, which subsequently allowed functional recovery of thalamo-cortical connectivity reflected in electrophysiological improvements. TH provided a similar degree of histological protection in the thalamus; however, its effects on cortical function may arise through distinct mechanisms such as reduced cerebral metabolism, decreased excitotoxicity, and inhibition of seizure activity [43]. TH effectively suppressed seizures in our current study, likely due to its known anticonvulsant mechanisms, including reduced cerebral metabolism, decreased glutamate release, and inhibition of excitatory synaptic transmission [17,44]. The observed delay in the onset of seizures with H_2_ treatment may reflect its ability to attenuate oxidative stress, suppress neuro-inflammation, and preserve mitochondrial and membrane integrity in HI brain injury, thereby raising the threshold for neuronal excitability [24,45]. However, the total seizure burden (detected length of abnormal EEG activity) was not significantly reduced by H_2_ treatment; in this aspect, the anticonvulsive actions of TH appears to be superior.

The model used in the present study has important limitations. It did not allow the exploration of potential sex-specific differences in response to the applied neuroprotective treatments, did not include sham-control groups relying on previously performed control experiments, and it was not appropriate to establish if TH and H_2_ can be synergistic in their neuroprotective effects. Indeed, these limitations can be overcome when designing future studies as H_2_ treatment appears not to affect physiological parameters or even preserve cardiovascular function and may therefore represent a safe and practical adjunct or even an alternative to TH.

## 5. Conclusions

We conclude that the asphyxia protocol used in the present study elicits a unique neuronal injury pattern, namely severe neuronal injury that was virtually restricted to the thalamus. In this HIE model, 48 h inhalation of neuroprotective concentration of H_2_ elicited significant thalamic neuroprotection that is comparable to that of TH. Although the neuropathology findings do not allow testing of synergism between the two treatments, the electrophysiological findings suggest that the two treatments may trigger complementary actions shown by differences in the occurrence of seizures and the complexity of the recovered EEG activity. These findings highlight the potential of H_2_ as a safe adjunct or alternative to TH in neonatal HIE and underscore the need for further mechanistic studies to optimize translational strategies.

## Data Availability

The original contributions presented in this study are included in the article/Supplementary Material. Further inquiries can be directed to the corresponding authors.

## Supplementary Materials

The following supporting information can be downloaded at https://www.mdpi.com/article/10.3390/antiox14121405/s1, Figure S1: Spectral power analysis of EEG signals in asphyxiated piglets treated with normothermia (A-NT), hydrogen ventilation (A-H_2_), or hypothermia (A-HT). Spectral power values (in µV^2^/Hz) were calculated from representative EEG recordings. No significant differences in total spectral power were observed between the groups. Data are shown as mean ± SEM.

## Abbreviations

The following abbreviations are used in this manuscript:

A/ASPH	asphyxia
B	baseline
EEG	electroencephalography
FFT	fast Fourier transform
FiO_2_	fraction of inspired oxygen
H&E	hematoxylin–eosin
HI	hypoxic-ischemic
HIE	hypoxic–ischemic encephalopathy
HR	heart rate
InstSpEnt	Instantaneous Spectral Entropy
MABP	mean arterial blood pressure
PSD	power spectral densities
ROS	reactive oxygen species
RR	respiratory rate
SEF	spectral edge frequency
SEM	standard error of the mean
spO_2_	oxygen saturation
TH	therapeutic hypothermia
VEP	visual evoked potential
