# Supplementary material for: Molecular Hydrogen Affords Similar Neuroprotection to Therapeutic Hypothermia in a Porcine Model of Neonatal Hypoxic–Ischemic Encephalopathy
Source: Antioxidants (Basel). 2025 Nov 25;14(12):1405. doi: 10.3390/antiox14121405 (PMC12729368; doi:10.3390/antiox14121405)
Supplement: Supplementary file 1 [file antioxidants-14-01405-s001.zip › antioxidants-3918400-supplementary.pdf]

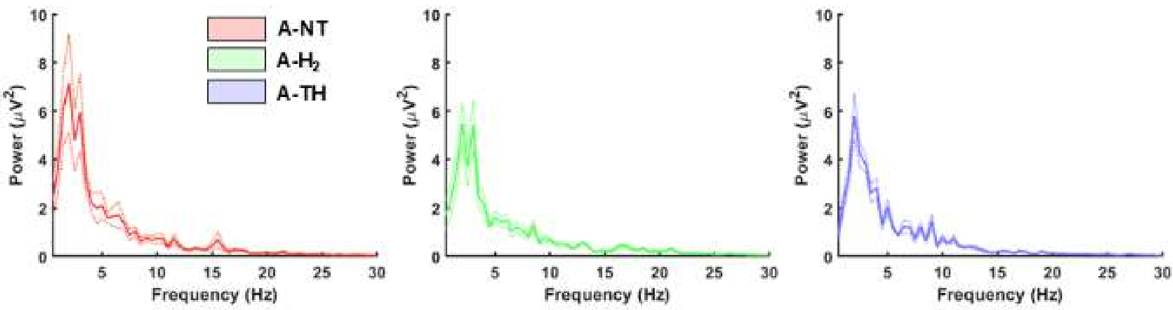

Figure S1. Spectral power analysis of EEG signals in asphyxiated piglets treated with normothermia (A-NT), hydrogen ventilation (A-H_2), or hypothermia (A-HT). Spectral power values (in $\mu V^2/Hz$) were calculated from representative EEG recordings. No significant differences in total spectral power were observed between the groups. Data are shown as mean ± SEM.
